# The Role of Stressful Childhood Experiences in Shaping Later-Life Memory Loss Among Black and White U.S. Adults

**DOI:** 10.1093/geroni/igaa057.948

**Published:** 2020-12-16

**Authors:** Caroline Hartnett

**Affiliations:** University of South Carolina, Columbia, South Carolina, United States

## Abstract

Cognitive decline common in the U.S. and greatly impacts quality of life, both for those who experience it and for those who care for them. Black Americans experience higher burdens of cognitive decline but the mechanisms underlying this disparity have not been fully elucidated. Stress experienced in early life is a promising explanatory factor, since stress and cognition are linked, childhood stressors been shown to have a range of negative implications later in life, and Black children experience more childhood stressors than White children, on average. In this paper, we use data from the Behavioral Risk Factor Surveillance System (BRFSS) to examine whether stressful experiences in childhood help explain Black-White disparities in memory loss. These data were available for 5 state-years between 2011 and 2017 (n=11,708). Preliminary results indicate that, while stressful childhood experiences are strongly associated with memory loss, stressful experiences do not mediate the association between race and memory loss. However, race does appear to moderate the association between stressful childhood experiences and memory loss. Specifically, stressful experiences are associated with a higher likelihood of memory loss for Black adults compared to White adults.In addition, there seem to be some noteworthy patterns across different types of experiences (i.e. parental drinking may predict later memory loss more strongly for Black adults than White adults, but parental hitting may predict memory loss more strongly for White adults than Black adults).

